# Diagnostic Accuracy of B-Mode- and Contrast-Enhanced Ultrasound in Differentiating Malignant from Benign Pleural Effusions

**DOI:** 10.3390/diagnostics11071293

**Published:** 2021-07-19

**Authors:** Ehsan Safai Zadeh, Johanna Weide, Christoph Frank Dietrich, Corinna Trenker, Andreas Rembert Koczulla, Christian Görg

**Affiliations:** 1 Interdisciplinary Center of Ultrasound Diagnostics, University Hospital Giessen and Marburg, Philipps University Marburg, Baldingerstraße, 35033 Marburg, Germany; ehsan_sz@yahoo.de (E.S.Z.); johannaweide@outlook.de (J.W.); 2 Department Allgemeine Innere Medizin (DAIM), Kliniken Hirslanden Bern, Beau Site, Salem und Permanence, 3018 Bern, Switzerland; c.f.dietrich@googlemail.com; 3 Haematology, Oncology and Immunology, University Hospital Giessen and Marburg, Philipps University Marburg, Baldingerstraße, 35033 Marburg, Germany; trenker@med.uni-marburg.de; 4 Institute for Pulmonary Rehabilitation Research, Schoen Klinik Berchtesgadener Land, 83471 Schoenau am Koenigssee, Germany; koczulla@med.uni-marburg.de; 5 Department of Pulmonary Rehabilitation, Philipps-University of Marburg, German Center for Lung Research (DZL), 35033 Marburg, Germany; koczulla@med.uni-marburg.de; 6 Gastroenterology, Endocrinology, Metabolism and Clinical Infectiology, University Hospital Giessen and Marburg, Philipp University of Marburg, Baldingerstraße, 35033 Marburg, Germany

**Keywords:** ultrasound, CEUS, pleural effusions, cytology, diagnosis

## Abstract

Purpose: To evaluate the value of CEUS in differentiating malignant from benign pleural effusions (PEs). Methods: From 2008 to 2017, 83 patients with PEs of unknown cause were examined using B-mode thoracic ultrasound (B-TUS), CEUS, and cytological examination. The extent of enhancement of the pleural thickening, the presence of enhancement of septa or a solid mass within the PE, and the homogeneity of the enhancement in the associated lung consolidation, were examined. Subsequently, the diagnostic value of cytology, B-TUS, and CEUS in differentiating malignant from benign PEs was determined. Results: With CEUS, markedly enhanced pleural thickening and inhomogeneous enhanced lung consolidation were significantly more frequently associated with malignancy (*p* < 0.05). In the subgroup analysis, the use of CEUS increased the sensitivity from 69.2 to 92.3 in patients with initial negative cytology but clinical suspicion of malignant PE; it also increased the specificity from 63.0 to 90.0, the positive predictive value from 69.2 to 92.3, the negative predictive value from 63.0 to 90.0, and the diagnostic accuracy from 66.7 to 87.5, in the evaluation of PE malignancy. Conclusion: The use of clinically based B-TUS and CEUS as a complementary method to cytological evaluation may be beneficial for evaluating a PE of unknown cause. CEUS patterns of enhanced pleural thickening and inhomogeneous enhanced lung consolidation may suggest a malignant PE.

## 1. Introduction

B-mode thoracic ultrasound (B-TUS) plays an important role in the assessment of pleural and pulmonary pathologies [1]. Due to its high sensitivity, absence of radiation exposure, and easy availability, B-TUS is the method of choice for detecting pleural effusions (PEs) [1,2]. Pleural effusions have many different causes, which may be benign or malignant [3]. For malignant PEs, the cytological examination obtained by ultrasound-guided thoracocentesis is the key to diagnosis, but it has variable sensitivity from 45.5% to 87.9%, depending on the type of disease [4]. Therefore, repeated specimen collection, invasive procedures such as pleural biopsy, and, ultimately, thoracoscopy are recommended for improving the diagnosis if the first specimen does not provide a definitive diagnosis [5,6,7]. However, all these procedures are invasive and may be associated with complications, such as pneumothorax and bleeding [8]. For this reason, additional imaging methods have been introduced to clarify the cause of the PE [9,10].

In computed tomography (CT), the presence of pleural thickening, nodular formations on the pleura, and an irregular pleura are considered reliable criteria for differentiating between benign and malignant PEs, and they can be helpful for directing imaging-guided pleural biopsy [10,11]. In a recent study, B-TUS was able to differentiate between transudate and exudative PEs [12]. It was shown that a complex septated effusion is indicative of an exudative PE [12]. Görg et al. proved in 1997 that, with B-TUS, the detection of pleural masses could be considered characteristic of a malignant effusion [9]. Despite its high specificity, B-TUS shows low sensitivity in detecting malignant PEs [13]. One of the methods to improve the sensitivity of B-TUS for the detection of malignant thoracic pathology is the use of contrast-enhanced ultrasound (CEUS) [14]. This helps to visualise different vascularisation patterns of pleural thickening, as well as focal lesions in PE-associated lung lesions; therefore, it can be helpful in differentiating between malignant and benign PEs [15,16].

The aim of this study was to evaluate the value of CEUS as a complementary method to improve the diagnostic accuracy of B-TUS in combination with the cytological examination.

## 2. Materials and Methods

The study was performed between March 2008 and December 2017 in a university hospital in Germany. All study patients were referred to the Interdisciplinary Centre of Ultrasound Diagnostics for the investigation of an unclear PE and exclusion of other thoracic pathologies. The inclusion criteria were: (1) a PE of unknown cause after an initial assessment, (2) completed B-TUS and CEUS examinations, (3) simultaneous ultrasound-guided thoracocentesis with the cytological examination, and (4) clinical follow-up for 6 months. A total of 83 patients met the entry criteria and were included in the study. All specimens were cytologically examined at the local pathology institute. The final PE diagnosis was documented in the clinical records based on repetitive thoracocentesis, histological data, and a clinical course of up to 6 months. The gold standard for all computations was the final diagnosis and results of the 6-month follow-up.

This retrospective study was approved by the local ethics committee and conducted in accordance with the amended Helsinki Declaration on the ethical principles for medical research involving human subjects. Informed consent was obtained from each patient for the ultrasound examinations.

### 2.1. Ultrasound Examinations

The B-TUS examinations were performed with an ACUSON SEQUOIA 512 GI ultrasound machine (Siemens, Erlangen, Germany). A 4C1 curved array transducer with frequencies between 3.5 and 4.5 MHz was used. The CEUS examinations were performed with the same transducer in 1.5 MHz contrast-specific mode according to the EFSUMB guidelines [17]. A bolus injection of 2.4 mL of SonoVue^®^ (Bracco Imaging S.p.A., Milan, Italy) contrast medium was given via peripheral venous access, followed by 10 mL NaCl 0.9%. During the first 30 s, PEs and thoracic pathologies were continuously examined. Subsequently, several short examinations in 30 s intervals, up to 3 min, were conducted. The pathologies presented in CEUS imaging were saved as images. All the patients were examined in an upright sitting position. All the ultrasound examinations were performed standardised and prospective by a German Society for Ultrasound in Medicine (DEGUM)-qualified Level-III examiner (C.G.). The ultrasound data were obtained during general clinical procedures and according to the hospital’s guidelines.

The following B-TUS data and CEUS parameters were retrospectively analysed by two independent, experienced investigators (E.S., C.G.). In the event of a discrepancy between the two investigators, the final decision was made by a third experienced investigator (C.T.). Cohen’s kappa statistics were applied to measure interrater reliability.

### 2.2. B-Mode Lung Ultrasound Parameters

1. The presence of pleural thickening (>3 mm), classified as nodular or flat thickening.
2. The pleural-effusion volume, classified as >1000 mL or <1000 mL. The volume was measured using the following formula: Pleural effusion volume (mL) = 70 × (basal lung-diaphragm distance (cm) + max. effusion height (cm)) [18].
3. The presence of a septated PE or a solid mass within the PE.
4. The homogeneity of the associated pulmonary consolidation, classified as homogeneous or inhomogeneous.

### 2.3. Contrast-Enhanced Ultrasound Parameters

1. The extent of enhancement of the pleural thickening in the arterial phase was classified as reduced or absent and marked enhancement. Splenic enhancement was used as an in vivo reference for comparison [16,19].
2. The presence of enhancement of septa or a solid mass within the PE [16].
3. The homogeneity of enhancement in the associated lung consolidation was classified as homogeneous or inhomogeneous. Inhomogeneous enhancement was defined as a perfused lesion with coexisting non-perfused or reduced perfused areas [15,20].

### 2.4. Statistical Analysis

Statistical evaluation was conducted on the categorical variable with Fisher’s exact test and on the continuous data with Mann–Whitney tests. Cohen’s kappa statistics were applied to measure interrater reliability, and a *p*-value < 0.05 was defined as significant. In addition, sensitivity, specificity, positive and negative predictive values, and diagnostic accuracy were evaluated for the cytological examination and the pathological ultrasound findings associated with malignancy.

## 3. Results

### 3.1. Characteristics of the Participants

Of the 83 patients, 60 were men and 23 were women. The average age was 62.2 years, with a range of 21–91. The final clinical diagnosis was malignant PE in 42/83 (50.6%) of the patients and benign PE in 41/83 (49.4%). There was no significant difference between patients with benign and malignant PEs in terms of age or gender (*p* > 0.05).

The final causes of PEs in all the patients are shown in Table 1.

### 3.2. Initial Cytopathological Data of the Patients

On the initial cytopathological examination, malignant cells in the effusions were detectable in *n* = 29 (34.9%) of the patients, and these effusions were classified as malignant PEs. In 54 (65.1%) of the patients, an initial cytopathological examination showed no malignant cells. Regarding the final cause of PE, the initial cytopathological examination showed a sensitivity of 69.0%, specificity of 100%, positive predictive value of 100%, negative predictive value of 75.9%, and diagnostic accuracy of 84.3% for identifying a malignant PE.

**Figure 1:**
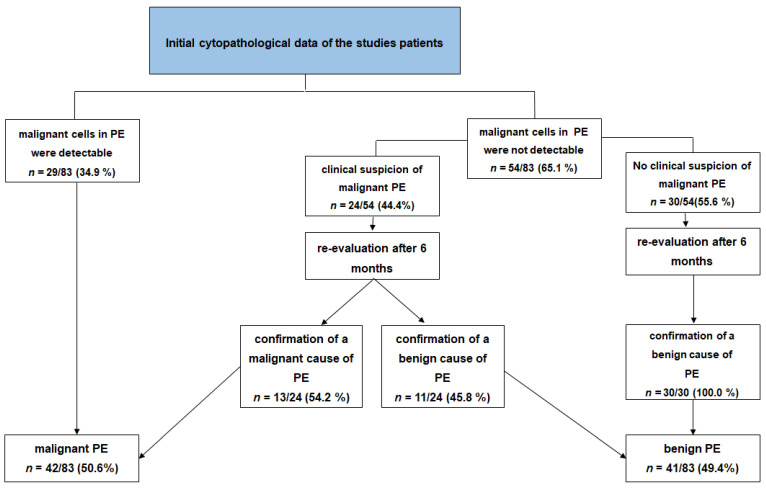
Work-up to the final diagnosis of PE in 83 patients with respect to the initial cytopathological results, clinical background, and follow-up.

Among the patients with negative initial cytology, 24/54 (44.4%) cases had a clinical suspicion of malignant PE, despite this initial result. In the re-evaluation of clinical data after 6 months, the PE was secondarily classified as malignant in 13/24 (54.2%) of the patients through a histopathological examination after either a pleural (7/13; 53.8%) or a pulmonary biopsy (6/13; 46.2%). In 11/24 (45.8%) of the patients with an initial benign cytology but suspected malignancy, a final benign PE was diagnosed by a clinical follow-up and confirmation of a benign cause (six parapneumonic, four congestive heart disease, and one non-infectious granulomatous disease). In 30/54 (55.6%) of the patients, the PE was initially classified as benign, in accordance with their “benign” clinical background. In the re-evaluation of clinical data after 6 months, all these PEs were confirmed to be truly benign. Figure 1 presents the work-up to the final diagnosis of PE with respect to the initial cytopathological results, clinical background, and follow-up.

### 3.3. Ultrasound Data

There was a good agreement between the examiners for the B-TUS and CEUS parameters (Cohen’s kappa = 0.78).

### 3.4. B-Mode Thoracic Ultrasound

The diagnostic data from the B-TUS examination are summarised in Table 2.

**Figure 2:**
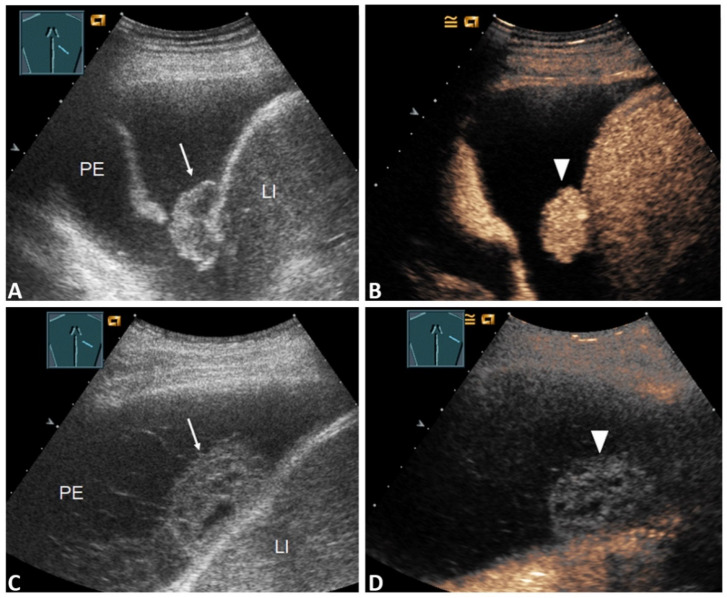
(**A**) 59-year-old patient with a right-sided PE. The effusion cytology was negative. B-TUS shows conspicuous nodular pleural thickening (arrow); (**B**) In the CEUS images, the nodular pleural thickening shows marked enhancement, such as in metastasis (stick). The final diagnosis was a malignant PE based on histologically confirmed pulmonary sarcoma. LI = Liver. (**C**) 64-year-old patient with fever, dyspnea, and a right PE. Effusion cytology was negative. B-US shows a septated effusion with a nodular formation on the diaphragm (arrow); (**D**) CEUS shows nodular pleural thickening with no enhancement, similar to fibrin bodies (sticks). The final diagnosis was a benign parapneumonic PE. LI = Liver.

**Figure 3:**
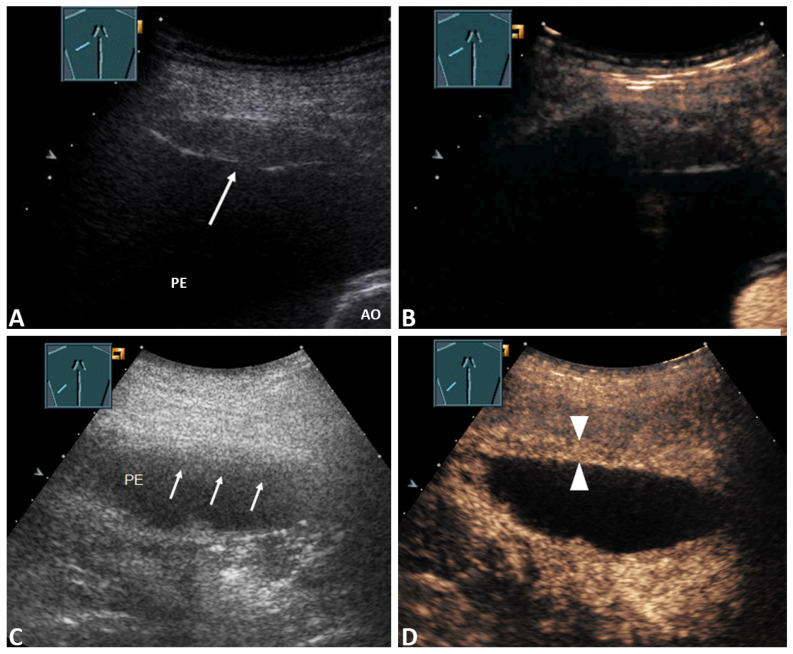
(**A**) 74-year-old patient with decompensated heart failure and a right-sided PE. The effusion cytology is negative. Pleural thickening is conspicuous in the B-TUS images (arrow); (**B**) CEUS shows pleural thickening with no enhancement; the final diagnosis was a benign PE with pleural thickening, based on histologically confirmed fibrinous pleuritis. AO = Aorta. (**C**) 75-year-old patient with chronic obstructive pulmonary disease, fever, and a left-sided narrow PE. The effusion cytology was negative. B-US shows conspicuous fuzzy pleural thickening (arrows); (**D**) CEUS reveals sharp pleural thickening with marked enhancement (sticks). The final diagnosis was a benign PE due to empyema.

In terms of the presence of a septated PE or a solid mass in the PE and inhomogeneous lung consolidation in the B-TUS images, there were no significant differences between patients with a malignant PE and those with a benign PE (*p* > 0.05, Fisher’s exact test). The presence of a PE with a volume > 1000 mL and pleural thickening was significantly more frequently associated with malignancy (*p* < 0.05, Fisher’s exact test). Among the patients with pleural thickening (28/83; 33.7%), 12/28 (42.9%) revealed nodular (Figure 2A,C), and 16/28 (57.1%) had flat thickening of the pleura (Figure 3A,C).

In those with nodular pleural thickening, 11/12 (91.7%) cases had malignant PEs, and those with flat pleural thickening had malignant PEs in 9/16 (56.3%) of the cases.

### 3.5. Contrast-Enhanced Thoracic Ultrasound

In total, using CEUS, a pleural thickening was detected in *n* = 33/83 (39.8%) study patients, and no pleural thickening was detected in *n* = 50/83 (60.2%). In *n* = 27/33 (81.1%) patients, a pleural thickening with marked enhancement was seen. Furthermore, in *n* = 6/33 (18.9%) patients, a pleural thickening with reduced or absent enhancement was observed. Using CEUS, in total, five more flat pleural thickenings and 13 more inhomogeneous pulmonary consolidations were detected in comparison with B-TUS. Regarding nodular pleural thickening or effusion pathologies (septa), there was no difference in the visualisation of pathologic structures between B-TUS and CEUS. Further details concerning the diagnostic data from the CEUS examination are summarised in Table 3.

**Table 3:** CEUS data of the *n* = 83 patients, subdivided between final malignant and benign PE.

Variable	All Patients *n* = 83	Malignant PE *n* = 42	Benign PE *n* = 41	*p*-Value
Pathological ultrasound findings with CEUS				
Pleural thickening with marked enhancement (Figure 2B and Figure 3D)	27 (32.5%)	20 (47.6%)	7 (17.1%)	0.005 *
Pleural thickening with absent or reduced enhancement or no pleural thickening (Figure 3B)	56 (67.5%)	22 (52.4%)	34 (82.9%)	
Enhancement of septa or solid formation (Figure 4D)	2 (2.4%)	2 (4.8%)	0 (0.0%)	0.494 *
No enhancement of septa or solid formation (Figure 5C)	81 (97.6%)	40 (95.2%)	41(100.0%)	
Inhomogeneous enhancement of lung consolidation (Figure 5B–D)	25 (30.1%)	19 (45.2%)	6 (14.6%)	0.004 *
Homogeneous enhancement of lung consolidation	58 (69.9%)	23 (54.8%)	35 (85.4%)	

The values are indicated as number (%). A *p*-value < 0.05 was defined as significant. *: Fisher’s exact test.

Among those with a marked enhancement of pleural thickening (27/83; 32.5%), *n* = 11/27 (40.7%) patients revealed nodular, and *n* = 16 (59.3%) had flat thickening of the pleura. Of the patients with nodular pleural thickening and marked enhancement, all 11/11 (100.0%) cases had a final malignant PE (Figure 2A,B). Of those with flat pleural thickening, *n* = 9/16 (56.3%) had a final malignant PE. In all patients with benign PEs, flat pleural thickening, and marked enhancement (7/7; 100%), the underlying disease was pleural empyema, as shown in Figure 3D. In those with pleural thickening and absent or reduced enhancement, *n* = 5/6 (83%) patients had a benign final diagnosis (Figure 3B), and in *n* = 1/6 (17%) a malignant PE was cytologically confirmed.

In total, using CEUS, an inhomogeneous enhancement of the lung consolidation was detected in *n*= 25/83 (30.1%) study patients. Inhomogeneous enhancement of the lung consolidation with a final malignant PE was found in 19 patients (Figure 5B).

**Figure 4:**
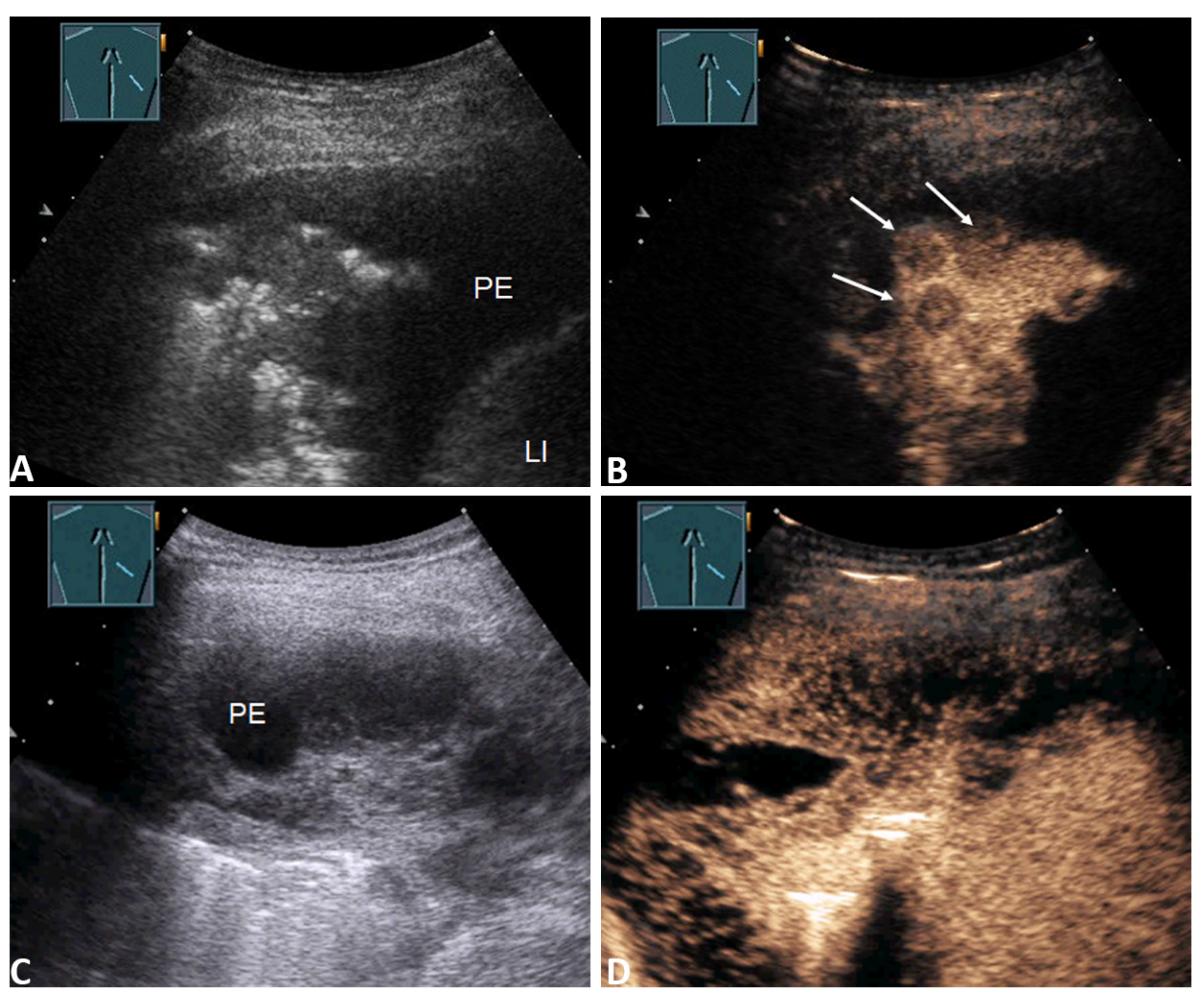
(**A**) 55-year-old patient with a right-sided PE. Effusion cytology was negative. B-TUS shows inhomogeneous lung consolidation; (**B**) In the CEUS images, the lung consolidation shows hypoechoic round lesions such as metastases (arrows). The final diagnosis was a malignant PE based on histologically confirmed metastatic adenocarcinoma of the oesophagus. LI = Liver. (**C**) 55-year-old patient with a right-sided PE. Effusion cytology was positive. B-TUS shows a polyseptated effusion; (**D**) CEUS shows marked enhancement of the septa. The final diagnosis was a malignant effusion due to mantle cell lymphoma with lung involvement.

The causes were primary bronchial carcinoma in 10/19 (52.6%) patients, pulmonary metastasis of solid carcinomas in 6/19 (31.6%) patients (Figure 4B), and pleural mesothelioma in 3/19 (15.8%) patients.

**Figure 5:**
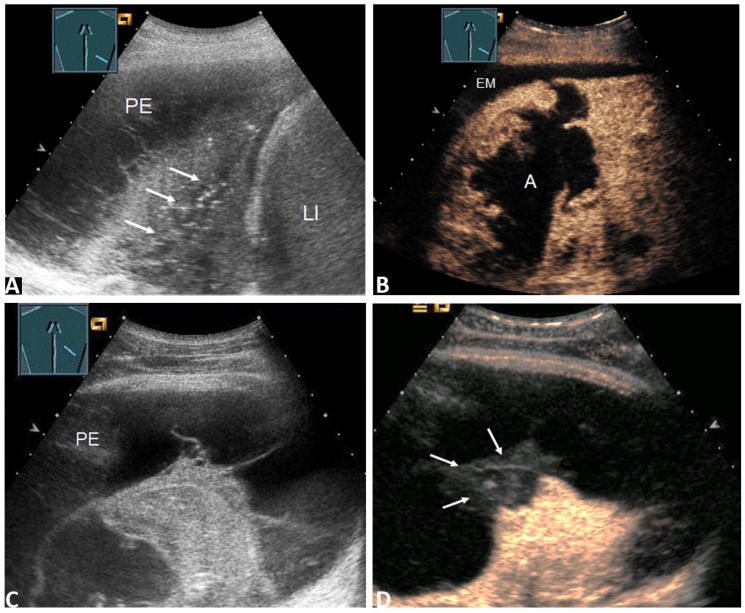
(**A**) 68-year-old patient with a right lateral PE. Effusion cytology was negative. B-TUS shows a PE with septa and inhomogeneous lung consolidation (arrows); (**B**) In the CEUS images, the lung consolidation shows a large anechoic liquidation, such as in cases of abscess or necrosis. The final diagnosis was a malignant PE due to central non-small cell lung carcinoma (NSCLC) with consecutive lung abscess (A) and pleural empyema (EM). LI = Liver. (**C**) 27-year-old patient with fever and a right-sided PE. Effusion cytology was negative B-TUS shows a PE with septation and lung consolidation; (**D**) CEUS shows consolidation with inhomogeneous enhancement and a peripheral non-enhanced wedge-shaped area similar to a pulmonary infarction (arrows). The final diagnosis was a benign PE with a pulmonary artery embolism morphologically confirmed through CT pulmonary angiography and histologically confirmed infarct pneumonia.

Inhomogeneous enhancement of the lung consolidation with a benign PE was found in six patients. The causes were an inflammatory process (parapneumonic, non-infectious granulomatous diseases and IgG4-related pleurisy) for 3/6 (50%) of the patients and pulmonary artery embolism for the other 3/6 (50%; see Figure 5D).

From the CEUS images, marked enhancement of the pleural thickening (Figure 2B) and inhomogeneous enhancement of the lung consolidation (Figure 5B) were significantly more frequently associated with a malignant PE (*p* < 0.05, Fisher’s exact test).

Furthermore, there were no significant differences between patients with a malignant PE and those with a benign PE in terms of the presence of septal enhancement or a solid mass in the PE. However, all the PEs with the presence of septa enhancement were malignant (2/2; 100%), as shown in Figure 4D and Table 3.

Data from the diagnostic accuracy of the initial cytopathological examination, B-TUS, and CEUS for all the patients in terms of sensitivity, specificity, and positive and negative predictive value are summarised in Table 4.

### 3.6. Negative Cytology and Clinical Suspicion

The 24 patients with an initial negative cytopathological examination but clinical suspicion of malignant effusion were investigated in a subgroup analysis (Figure 1).

Within this subgroup, the presence of one or more parameters associated with malignancy in the B-TUS images showed a sensitivity of 69.2%, specificity of 63.4%, positive predictive value of 69.2%, negative predictive value of 63.4%, and diagnostic accuracy of 66.7% for the identification of a malignant PE (Table 5).

The presence of one or more parameters associated with malignancy in the CEUS images had a sensitivity of 92.3%, specificity of 90.0%, positive predictive value of 92.3%, negative predictive value of 90.0%, and diagnostic accuracy of 87.5% for the identification of a malignant PE (Table 5).

## 4. Discussion

For the evaluation of unclear PE, contrast-enhanced CT is recommended as the method of choice in the guidelines from the British Thoracic Society [21]. In addition to contrast-enhanced CT, B-TUS is widely used as a cost-effective method without radiation exposure for the evaluation of PE [1,9,15]. However, only limited data are available regarding the diagnostic performance of CEUS as an additional imaging modality in the diagnosis of unclear PE [16].

In this retrospective study, we investigated the diagnostic accuracy of B-TUS and CEUS for 83 patients with PEs of unknown cause. Furthermore, we investigated the diagnostic value of B-TUS and CEUS for evaluating PE malignancy in all the patients, as well as in a subgroup of 24 with initial negative cytology and clinical suspicion of malignant PE.

Regarding B-TUS patterns, we found that the detection of a PE volume > 1000 mL and pleural thickening was significantly more often associated with malignant effusion. From the CEUS images, pleural thickening with marked contrast enhancement and inhomogeneous enhancement of the lung consolidation was significantly more often associated with malignancy. In this study, the PEs were malignant in all 11/11 (100%) cases with nodular pleural thickening and marked enhancement. Interestingly, 6/11 (54.5%) of the effusions in these patients were classified as benign in the initial cytopathological examination. Based on these findings, nodular pleural thickening with marked enhancement should be considered malignant, even if there is no indication of malignancy in cytopathological examinations.

For all the patients in the study, the diagnostic accuracy was 63.9% for B-TUS and 72.3% for CEUS in terms of evaluating malignancy. The diagnostic performance of B-TUS and CEUS in the present study compared with the findings of previously performed B-TUS and contrast-enhanced CT studies is summarised in Table 6.

The higher diagnostic performance in the two previously performed B-TUS studies may be due to the inclusion criteria, the differences in patient spectrums, and the examination criteria in these studies. In both studies, only patients with suspicion of malignant effusion were included [22,23]. Furthermore, both studies considered the presence of hepatic metastases as an indication of malignant PE [22,23]. In the study by Qureshi et al., all patients with clinical or radiological features of empyema were excluded [22]. This may have resulted in selection bias and could explain the higher specificity and positive predictive value compared with the findings of the current study.

The two previously performed contrast-enhanced CT studies showed higher sensitivity and specificity compared with CEUS in the diagnosis of malignant PE [10,24]. Compared with ultrasound, CT provides a better overview and can visualise more areas of the thorax. In ultrasound, due to air and the bony structures of the thorax, only approximately 70% of the pleura can be visualised and examined [25]. This could be the reason for the higher diagnostic performance of CT compared with CEUS.

In contrast to the results for the patients in the overall study, the subgroup analysis of those with an initial negative cytology and clinical suspicion of malignant PE revealed a better diagnostic value for CEUS compared to B-TUS regarding the evaluation of malignancy. The use of CEUS increased the sensitivity in the subgroup from 69.2 to 92.3, the specificity from 63.0 to 90.0, the positive predictive value from 69.2 to 92.3, the negative predictive value from 63.0 to 90.0, and the diagnostic accuracy from 66.7 to 87.5 for the evaluation of PE malignancy. In this group, CEUS showed high diagnostic performance and could be considered to be a useful imaging modality.

Pleural thickening occurs in both benign and malignant diseases [16], and it may be the result of an inflammatory process, which is indicated with a marked enhancement of pleural thickening in the CEUS images [26,27]. In this study, the causes of all pleural thickenings with marked enhancement were malignant or inflammatory processes due to pleural empyema. This finding can be used in combination with clinical suspicion to improve the identification of malignant PEs in patients with initially negative cytological results. A further value of CEUS is the identification of disturbed perfusion of associated lung consolidation [20,28,29]. Inhomogeneous perfusion seen in the CEUS can be found in various pathological conditions, including peripheral pulmonary infarction, peripheral pulmonary granulomatous lesions [28], chronic pneumonia-associated lung abscess, and tumour-associated necrosis or vascularisation disturbance in the consolidated lung [15,29]. PE-associated lung consolidations with peripheral wedge-shaped non-perfused areas are highly suggestive of infarction in patients with pulmonary embolic disease [20,30]. Pneumonia associated lung lesions may indicate lung abscesses with secondary development of parapneumonic empyema [15]. On the other hand, centrally located round lesions in the consolidated lung may indicate lung metastasis or necrosis [15]. These findings can be used in combination with clinical suspicion to improve the identification of the causes of PE. It should be highlighted that sonographic diagnosis is fundamentally based on clinical signs and symptoms. Finally, it should be mentioned that, in all detected pleural or parenchymatous lesions, CEUS-guided biopsy with histological examination is the method of choice to achieve the final diagnosis [31,32].

There are some limitations to this study. This study is limited by its retrospective nature. Furthermore, ultrasound examination is generally characterised by high interobserver variability. We could not blind the examiners to the study group, and blind interpretation of the ultrasound data by the ultrasound investigator was not possible. Furthermore, the study was performed on patients who were referred to the Interdisciplinary Centre of Ultrasound Diagnostics for the investigation of an unclear PE and exclusion of other thoracic pathologies and were investigated standardised by a single DEGUM-qualified Level-III examiner. Therefore, selection bias cannot be excluded. Finally, a pleural or a pulmonary biopsy with histological validation was not performed on all study patients.

### Interpretation

The findings of this study show that using clinically based B-TUS and CEUS as complementary methods to cytopathological examinations are beneficial for evaluating PEs of unknown causes. Furthermore, CEUS patterns of pleural thickening with marked enhancement and an inhomogeneous perfusion pattern of lung consolidation may suggest a malignant PE. However, for a definitive diagnosis of PE malignancy, repetitive cytology, clinical work-up, and, if necessary, a pleural or pulmonary histopathological examination is warranted.

## Figures and Tables

**Table 1:** Causes of PEs in *n* = 83 patients.

Cause of Benign PE	Number (%) of Patients	Cause of Malignant PE	Number (%) of Patients
Parapneumonic PE	18 (43.9)	Pulmonary metastasis of solid carcinomas	17 (40.5)
Pleura empyema	7 (17.1)	Primary bronchial carcinoma	17 (40.5)
Congestive heart failure	9 (22.0)	Pleural mesothelioma	4 (9.5)
Pulmonary artery embolism	3 (7.3)	Lung infiltration by malignant lymphoma	2 (4.8)
Non-infectious granulomatous diseases	2 (4.9)	Pulmonary metastasis of sarcoma	2 (4.8)
Liver cirrhosis	1 (2.4)	-	-
IgG4-related pleuritis	1 (2.4)	-	-
Total number of patients with benign PE	41 (100)	Total number of patients with malignant PE	42 (100)

The values are indicated as number (%). PE: pleural effusion.

**Table 2:** B-TUS data of the *n* = 83 patients, subdivided between final malignant and benign PE.

Variable	All Patients *n* = 83	Malignant PE *n* = 42	Benign PE *n* = 41	*p*-Value
**Pathological ultrasound findings with B-TUS**				
Pleural thickening	28 (33.7%)	20 (47.6%)	8 (19.5%)	0.010 *
No pleural thickening	55 (66.3%)	22 (52.4%)	33 (80.5%)	
PE volume ≤ 1000	51 (61.4%)	21 (50.0%)	30 (73.2%)	0.042 *
PE volume > 1000	32 (38.6%)	21 (50.0%)	11 (26.8%)	
Septa or solid formation in PE	24 (28.9%)	8 (19.0%)	16 (39.0%)	0.055 *
No septa or solid formation in PE	59 (71.1%)	34 (81.0%)	25 (61.0%)	
Inhomogeneous lung consolidation	12 (14.5%)	9 (21.4%)	3 (7.3%)	0.116 *
Homogeneous lung consolidation	71 (85.5%)	33 (78.6%)	38 (92.7%)	

The values are indicated as number (%). A *p*-value < 0.05 was defined as significant. *: Fisher’s exact test.

**Table 4:** Diagnostic performance of initial cytopathological examination, B-US, and CEUS for evaluating PE malignancy in *n* = 83 patients.

Variable	Sensitivity (%)	Specificity (%)	PPV (%)	NPV (%)	Diagnostic Accuracy (%)
Initial cytopathological examination	69.0	100.0	100.0	75.9	84.3
B-TUS data associated with malignancy (volume of PE > 1000 mL, pleural thickening)	69.1	58.5	63.0	64.7	63.9
CEUS data associated with malignancy (pleural thickening with marked enhancement and inhomogeneous enhancement of pulmonary consolidations)	73.8	70.7	72.1	72.5	72.3
B-TUS and CEUS data associated with malignancy	88.1	46.3	62.7	79.2	67.5

The values are indicated as number (%). PPV = positive predictive value; NPV = negative predictive value.

**Table 5:** Diagnostic performance of B-TUS and CEUS for evaluating PE malignancy in a subgroup of *n* = 24 patients with initial negative cytology and clinical suspicion of malignant PE.

Variable	Sensitivity (%)	Specificity (%)	PPV (%)	NPV (%)	Diagnostic Accuracy (%)
B-TUS data associated with malignancy (volume of PE > 1000 mL, pleural thickening)	69.2	63.4	69.2	63.4	66.7
CEUS data associated with malignancy (pleural thickening with marked and inhomogeneous enhancement of pulmonary consolidations)	92.3	90.0	92.3	90.0	87.5
B-TUS and CEUS data associated with malignancy	92.3	54.6	70.6	85.7	75.0

The values are indicated as number (%). PPV: positive predictive value; NPV: negative predictive value.

**Table 6:** Diagnostic performance of B-TUS, CEUS, and CECT for evaluating PE malignancy.

Imaging Modality	Cases	Year	Author	Sensitivity (%)	Specificity (%)	PPV (%)	NPV (%)	Diagnostic Accuracy (%)
B-TUS	52	2008	Qureshi et al. [22]	73	100	100	79	NS
B-TUS	133	2014	Bugalho et al. [23]	80.3	83.6	82.8	81.2	81.9
B-TUS	83	2021	Present study	69.1	58.5	63.0	64.7	63.9
CEUS	83	2021	Present study	73.8	70.7	72.1	72.5	72.3
CECT	40	2000	Traill et al. [10]	87.0	100	100	67.0	90.0
CECT	343	2015	Porcel et al. [24]	74.0	92.0	NS	NS	NS

B-TUS: B-mode thoracic ultrasound; CECT: contrast-enhanced computed tomography; CEUS: contrast-enhanced ultrasound; NPV: negative predictive value; NS: not specified; PPV: positive predictive value.

## Data Availability

The data presented in this study are available on request from the corresponding author.
